# Fabrication and Evaluation of a Flexible MEMS-Based Microthermal Flow Sensor

**DOI:** 10.3390/s21238153

**Published:** 2021-12-06

**Authors:** Myoung-Ock Cho, Woojin Jang, Si-Hyung Lim

**Affiliations:** 1 Department of Mechanical Systems Engineering, Graduate School, Kookmin University, Seoul 02707, Korea; myock@kookmin.ac.kr (M.-O.C.); jngwj2061@kookmin.ac.kr (W.J.); 2 School of Mechanical Engineering, Kookmin University, Seoul 02707, Korea

**Keywords:** MEMS, flexible thermal flow sensor, microheater, flow measurement

## Abstract

Based on the results of computational fluid dynamics simulations, this study designed and fabricated a flexible thermal-type micro flow sensor comprising one microheater and two thermistors using a micro-electromechanical system (MEMS) process on a flexible polyimide film. The thermistors were connected to a Wheatstone bridge circuit, and the resistance difference between the thermistors resulting from the generation of a flow was converted into an output voltage signal using LabVIEW software. A mini tube flow test was conducted to demonstrate the sensor’s detection of fluid velocity in gas and liquid flows. A good correlation was found between the experimental results and the simulation data. However, the results for the gas and liquid flows differed in that for gas, the output voltage increased with the fluid’s velocity and decreased against the liquid’s flow velocity. This study’s MEMS-based flexible microthermal flow sensor achieved a resolution of 1.1 cm/s in a liquid flow and 0.64 cm/s in a gas flow, respectively, within a fluid flow velocity range of 0–40 cm/s. The sensor is suitable for many applications; however, with some adaptations to its electrical packaging, it will be particularly suitable for detecting biosignals in healthcare applications, including measuring respiration and body fluids.

## 1. Introduction

The use of micro-electromechanical system (MEMS) technology satisfies various demands in the field of microsensors for small size, low power consumption, low cost, mass production, high accuracy, high sensitivity, etc. These advantages have led to multiple advances in the field of micro flow sensors [1]. Currently, MEMS-based micro flow sensors are found in various applications in industrial settings. The combination of MEMS technology and automatic control/AI technology is suitable for use in smart homes/green buildings, safety/environmental sensing, and healthcare fields [2], with the objective of realizing Internet of Things’ technology in relation to the construction of smart homes/green buildings. In addition, since it is also important to present a mathematical model in the design process prior to developing these MEMS devices, there was an effort to build a physical-mathematical model that is a basis for both presenting the operating conditions of the device and an objective explanation of the results. [3]. However, in the field of micro flow sensors, the most notable recent developments have been related to the healthcare sector and the measurement of biosignals, and many studies and advancements are currently underway in this area [4,5,6].

There are three main types of MEMS-based flow sensors: thermal flow sensors, piezoresistive flow sensors, and piezoelectric flow sensors. Of these, the simple structure and principle of the thermal flow sensor and its ability to measure a wide range of flow rates in both liquids and gases makes it popular in many different fields [7]. Three different thermal flow sensor methods can be used according to the means of measurement, and an appropriate technique can be selected according to the type of fluid and flow rate. The hot-wire method measures heat loss, the calorimetric method measures the thermal equilibrium between two thermistors, and the time-of-flight method measures the arrival time of heat pulses. In some studies, advanced sensor performance has been realized using a combination of these principles to maximize the advantages of each method [8,9,10,11]. 

To date, many different types of thermal flow sensors have been studied and developed, although few have progressed into commercial products. The majority of thermal flow sensors are used to measure the low flow rates of gases or liquids up to several milliliters/minute, and most reported data refer only to continuous flow rates. Preventing heat loss from a sensor by blocking the conduction of heat to a substrate is challenging for the thermal flow sensor. 

Ashauer et al. realized a response time of under 2 ms and a resolution of 0.1 mm/s in a thermal flow sensor across a wide range of flow rates by creating a thermally isolated structure using thermopiles on a silicon nitride membrane [12]. Dijkstra et al. implemented a calorimetric-based flow sensor with low hydraulic resistance that included a microchannel that was thermally isolated from the substrate with a channel height of several tens of micrometers to prevent any step coverage problems from occurring. The developed sensor detected a signal fluid flow as low as 300 nL/min [13]. Ahmed et al. developed a wireless, dual-mode, low-powered, calorimetric-based complementary metal–oxide semiconductor MEMS flow sensor to measure bidirectional gas [14,15].

The studies of micro thermal flow sensors for biomedical application are remarkable. Mistry and Mahapatra modeled a thermal flow sensor to develop a MEMS-based implantable sensor to monitor arterial blood flow and simulated the thermal distribution of the fluid flow [16]. Li et al. developed a smart catheter flow sensor to monitor cerebral blood flow; they used the constant temperature mode in the thermal diffusion flow measurement principle to establish a method for providing reliable data without drift [17]. Additionally, they used a compensation circuit system to solve the problem of continually increasing temperature in a medium. Jiang et al. developed a non-invasive respiratory monitoring system using a flexible hot-film smart sensing strip that was attached to a person’s upper lip to measure real-time respiration [18]. They used a Bluetooth module for wireless data acquisition and a smart device as a display module, which could be configured as a wearable device to monitor conditions such as sleep apnea. Okihara et al. fabricated a detachable flexible flow sensor to evaluate the partial respiration characteristics of the airway and measured the local flow [19]. The flexible sensors can be applied to the medical and industrial fields that require micro-flow measurement and analysis since they have a deformable property, which can access narrow and winding areas that are difficult to access with conventional sensors, and thus more accurate results can be expected. Finally, Hedrich et al. developed a MEMS-based thermal membrane anemometric flow sensor for connection to a breathing mask for long-term respiratory monitoring related to cardiovascular disease [20]. 

In this study, we developed a flexible microthermal flow sensor for simultaneously measuring high flow rates in gases and liquids from tens to hundreds of milliliters/minute using the MEMS process on a flexible substrate. It is anticipated that the sensor could be used in many fields for different purposes, including healthcare. Generally, the differential pressure meter, which is known to be suitable for high flow, has a limited dynamic range, and is also difficult to apply to a pulsating flow [12]. However, the present study’s flexible thermal flow sensor can measure high-flow fluids and has obtained meaningful results with pulsating liquid flows. In addition, the developed sensor can effectively reduce heat loss compared to metal or silicon-based sensors by using a polyimide film with a low thermal conductivity. This suggests that the developed sensor has the potential for future use in medical applications.

This study’s sensor electrode was designed with one central microheater and two thermistors—one on either side. It was developed by applying a calorimetric principle that measures the flow rate by detecting the change in the equilibrium state of both thermistors under a flow. A computational fluid dynamics (CFD) analysis determined the structure and size of the sensor, and the output voltage from the electrical circuit was designed and monitored in real time using LabVIEW software. The sensor signal was measured within a flow range of 25–200 sccm using N_2_ gas at 25–150 mL/min for a continuous liquid flow with a syringe pump. The signal was also verified in a pulsating liquid flow using an artificial heartbeat model within a flow range of 25–200 mL/min. A highly accurate result was obtained using the simplest type of bridge circuit based on the calorimetric principle; many studies use a complicated circuit to compensate for the signal drift phenomenon.

This study fabricated a MEMS-based flexible micro flow sensor and conducted flow velocity measurement tests on gases and liquids using a basic technique. Important experimental results were obtained within a range of sizes applicable to the human body. The future development of electrical packaging for the developed sensor can extend its application to the measurement of biosignals in the healthcare field, including for the measurement of respiration and body fluids.

## 2. Materials and Methods

### 2.1. Modeling and Simulation

**Figure 1:**
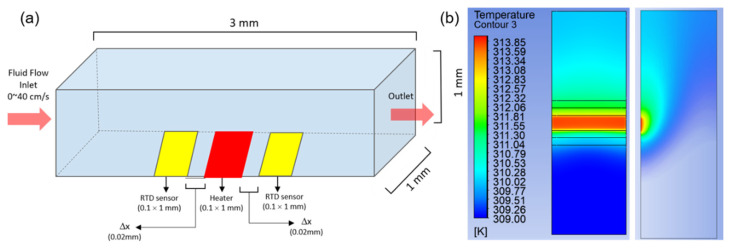
(**a**) Schematic design of the simulation area, the sensor electrode, and the direction of the fluid. (**b**) Result of the temperature distribution in the setting zone and the sensor electrode under a flow.

To review the anticipated difficulties in designing and manufacturing the flexible microthermal flow sensor and to predict the results, a CFD analysis was conducted using ANSYS 17.0 Fluent software before the development of the sensor [21]. For simplicity, the flow path was regarded as a rectangular pipe, and the physical properties of the fluids were established for gas (air) and liquid (blood), respectively. The study predicted the temperature change of the thermistors on both sides of the microsensor electrode resulting from the change in the fluid’s velocity. The microheater and thermistors comprising the sensor were set in platinum (Pt). Considering the size of the sensor to be developed, the size of the sensor’s electrode for the heater and the thermistors was 0.1 × 1.0 mm, and the electrode’s elements were separated from one side of the flow path using the “lines from sketches” menu. The ends of the flow path were defined as the inlet and outlet, respectively, and the fluid velocity was provided differentially at 10–40 cm/s at the inlet. The temperature of the microheater was set to 4 °C above that of the surrounding fluid, and the temperature difference between the two thermistors according to the fluid velocity was simulated. Figure 1a presents a schematic design of the simulation area setup, the size and arrangement of the sensor electrode, and the direction of the fluid, whereas Figure 1b shows the temperature distribution in the setting zone and the sensor electrode under flow conditions. The study optimized the distance between the heater and the thermistor by adjusting the distance between the two components and analyzing the linearity of the temperature difference according to the flow rate. Table 1 summarizes the shape and boundary conditions and the material properties used in the flow analysis.

### 2.2. Design and Fabrication of the MEMS Sensor Electrode

**Figure 2:**
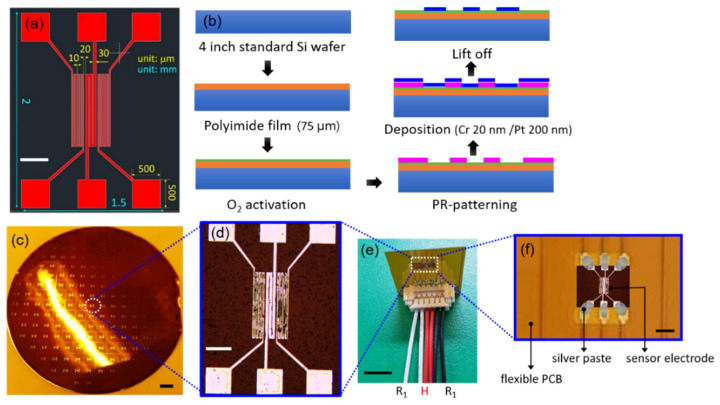
The designed and fabricated sensor electrode. (**a**) Final design of the sensor electrode, bar = 500 μm. (**b**) Schematic of the MEMS process to form the sensor electrode. (**c**,**d**) Sensor electrode fabricated by the MEMS process, bar = 6 mm and 500 μm, respectively. (**e**,**f**) Sensor electrode inserted in the thermal flow sensor module, bar = 5 mm and 1 mm, respectively.

This study used a flexible microthermal flow sensor in conjunction with a thermo-transfer method to measure the flow velocity in various fluids. The size and shape of the sensor were designed by establishing the correct temperature of the microheater and calculating the heat capacity of the sensor. The sensor shape was set to a structure in which the typical calorimetric metric principle was applied by arranging an upstream sensor and a downstream sensor on both sides of the central heater, and the size of each sensor electrodes were set to 0.1 by 1 mm in obedience to the simulation setting. Each electrode was in the form of a coil, and the central heater was set by calculating the width and length of the coil within the final width of 100 μm in consideration of the physical and thermal properties of the metal to meet the set temperature. The two thermistors were minimized in the width of the coil to increase the resistance so that a linear result could be expected by maximizing the temperature change, and the total width of the two thermistors did not exceed 100 μm, respectively. The final design of the sensor’s electrode is shown in Figure 2a. 

The dimensions of the sensor were approximately 1.3 mm (width) × 1.6 mm (length). To maximize the temperature difference between the two thermistors, the distance to the heater was minimized by placing the heater and the thermistors 20 μm apart. The sensor electrode was fabricated using a MEMS process based on a previously designed sensor structure. To ensure a thin, flexible sensor, the substrate was formed from a polyimide film rather than a general silicon wafer. First, a 75 μm thick polyimide film was laid on a silicon wafer, and O_2_ activation was performed to enhance the bonding with the photoresistor. Chrome (Cr) and Pt were sputtered at a thickness of 20 and 200 nm, respectively, and the sensor electrode was completed via a lift-off process. Figure 2b presents a schematic of the MEMS process for forming the sensor electrode, and Figure 2c,d shows the finished item. The completed sensor electrode demonstrated a resistance of approximately 100 Ω in the microheater and approximately 700 Ω in both thermistors.

### 2.3. Fabrication of the Sensor Module

**Figure 3:**
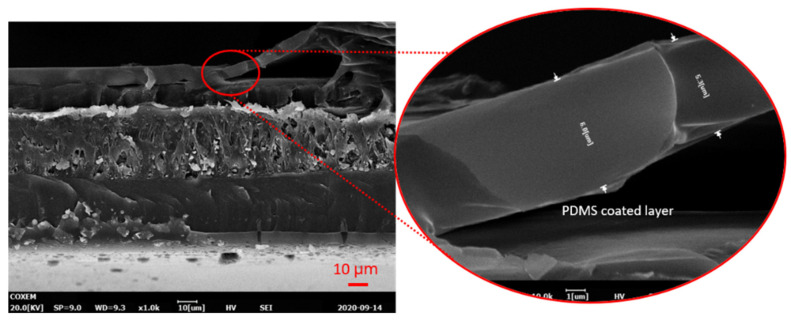
A scanning electron microscope (SEM) image of the polydimethylsiloxane (PDMS) layer on the flexible sensor substrate (cross-sectional view). The red circle shows a 10× magnified image of the PDMS layer.

A polyimide-based flexible printed circuit board (PCB) was fabricated to directly connect the MEMS Pt electrode to an electrical circuit. A section of electrode was cut and attached to the flexible PCB, and the connection pads on the PCB and the sensor electrode were connected using silver paste. The surface of the sensor module was double coated by immersion in a diluted PDMS solution to provide insulation while preserving high sensitivity. The thickness of the PDMS layer was determined via a scanning electron microscope (SEM) to be approximately 5–6 µm. A SEM image of the PDMS layer on the sensor module is presented in Figure 3. However, for the liquid flow test, the insulation layer was replaced with a coating of parylene-C since the former is more vulnerable to electrical short circuits, unlike a gas flow [22], and the thickness of the coating layer was 3 μm.

The sensor module was completed by attaching the pitch connector on the flexible PCB to the electrode sensor and connecting the jumper cables into it (Figure 2e,f). The microheater of the sensing unit was subjected to a heating test using the completed sensor module. A thermal imaging camera monitored the heat generated when a 1.25 V current was applied to the microheater via the sensor’s custom-made PCB.

### 2.4. Electronic Circuit System

**Figure 4:**
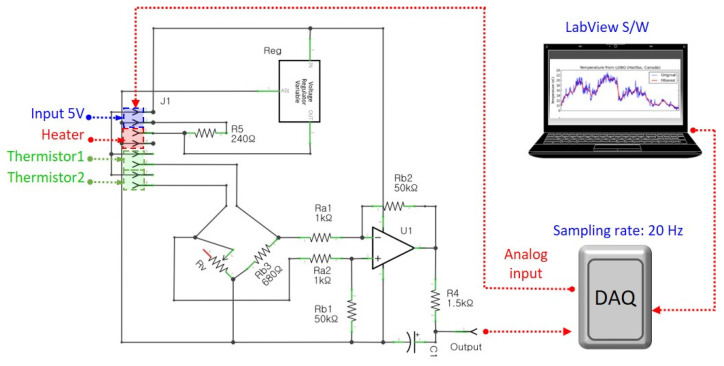
Circuit diagram for sensor driving and signal measurement workflow. The circuit system is powered by 5 V from the data acquisition (DAQ) device (myDAQ: NI, USA), and the output voltage is displayed via LabVIEW S/W (via DAQ).

This study designed an electrical circuit system, which included a Wheatstone bridge circuit for the sensor, and fabricated a 5 × 5 cm PCB. Figure 4 shows the circuit diagram and the signal measurement workflow. The two thermistors on either side of the microheater were connected to the Wheatstone bridge circuit, respectively. A voltage of 5 V was applied to the circuit from the DAQ, and a reduced voltage of 1.25 V was applied to the microheater via a regulator on the PCB. Under a flow, the temperature difference changes the resistance of the thermistors, which generates a voltage in the Wheatstone bridge circuit. The output voltage was amplified 50 times via a differential amplifier (LM358; National semiconductor, SantaClara, CA, USA), and the high-frequency noise was cut off by a resistor–capacitor (RC) filter. The output voltage signal that passed through the circuit system was digitized by DAQ before being analyzed and visualized in dedicated software. To analyze the signals of the flow sensor, this study constructed an analysis algorithm using LabVIEW software (LabVIEW, NI, Austin, TX, USA), and the voltage signal was acquired at a frequency of 20 Hz.

## 3. Results and Discussion

### 3.1. Visualization of the Temperature Changes by Simulation

This section uses computational simulations to predict the general performance and results of the detection of fluid flow using the fabricated sensor. Several factors to be considered when fabricating the sensor were checked in advance and were reflected in the design of the device. The simulation visualized the temperature difference between the two thermistors, and the variations provided the magnitude of the output voltage, which is directly or inversely proportional to the flow velocity depending on the properties of the fluid. According to the CFD analysis for the temperature difference between the thermistors of the sensor electrodes with respect to the flow rate of the fluid, in the case of gas, ΔT (Thermistor_Downstream_ − Thermistor_Upstream_) showed a tendency to increase as the velocity also increased. However, when the physical properties of the fluid were changed to those of blood (which is a liquid), there was a tendency for ΔT to decrease at speeds exceeding 1 cm/s.

Generally, in a calorimetric thermal flow sensor, the temperature of a downstream sensor is higher than that of an upstream one according to the heat provided by a heater. Accordingly, the temperature difference, ΔT, is defined as the value obtained by subtracting the upstream temperature from the downstream temperature. For gas, the temperature decrease of the upstream sensor according to the flow rate is greater than that of the downstream sensor; therefore, the ΔT increases according to the increase in flow rate. However, for liquid, there is a rapid temperature decrease for the upstream sensor under a flow; consequently, the additional temperature reduction according to the flow rate is much lower than the size of the temperature decrease in the downstream sensor. Therefore, the temperature difference between the two sensors is inversely proportional to the increase in flow rate. It is believed that this phenomenon is attributed to the difference in thermal conductivity between gas (air) and liquid (blood) of 0.025–0.03 and 0.52–0.6 W/m·k, respectively, representing a difference of approximately 17–20 times.

**Figure 5:**
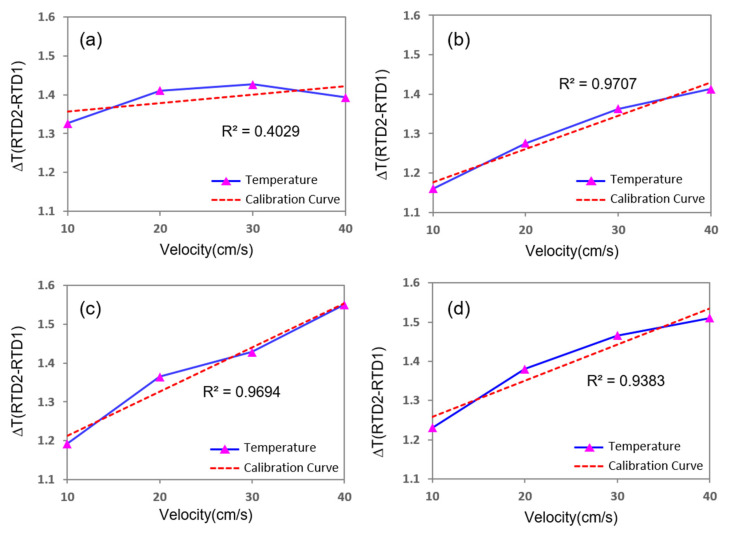
Simulation results for gas flow with respect to the distance between the microheater and thermistors. (**a**,**b**) Distance between the microheater and the electrode is symmetrically located by 0.2 and 0.1 mm increments, respectively. (**c**) Electrodes are located asymmetrically 0.08 mm for the sensor upstream to the heater and 0.1 mm for the sensor downstream to the heater. (**d**) Electrodes are located asymmetrically 0.1 mm for the sensor upstream to the heater and 0.09 mm for the heater downstream to the sensor.

**Figure 6:**
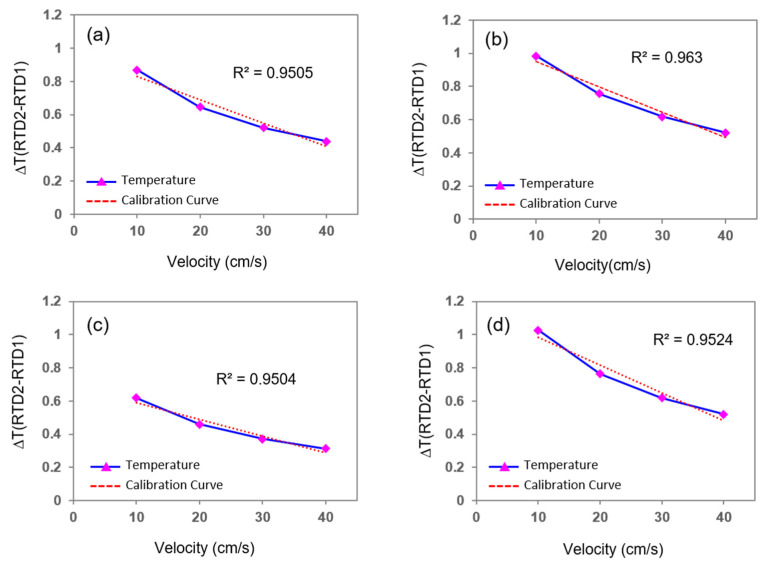
Simulation results for the liquid flow with respect to the distance between the microheater and thermistors. (**a**,**b**) Distance between the microheater and the electrode is located symmetrically by 0.05 mm and 0.02 mm increments, respectively. (**c**) Electrodes are located asymmetrically 0.02 mm for the sensor upstream to the heater and 0.05 mm for the sensor downstream to the heater. (**d**) Electrodes are located asymmetrically 0.05 mm for the sensor upstream to the heater and 0.02 mm for the downstream sensor to the heater.

Additionally, when the width of both thermistors and the microheater was 0.1 mm and the distance between the heater and thermistors was symmetrically 0.01–0.02 mm, the temperature difference between the two thermistors increased or decreased almost linearly with the change in the flow rate of the fluid. Figure 5 and Figure 6 present the simulation results in graph form for gas and liquid, respectively. When the velocity of the fluid increased by 10 cm/s, the temperature difference between the two thermistors increased by approximately 0.1 °C for gas, whereas it decreased by 0.2 °C for liquid.

### 3.2. Experimental Results for Gas Flow 

To evaluate the performance of the developed flexible thermal flow sensor, this study configured an experimental setup to detect the change in voltage according to the change in flow rate. The MEMS flow sensor module was inserted into 3 mm diameter tubing to locate the sensor electrode in the center of the tube, and the cables from the sensor were connected to the electrical circuit system outside the tubing. The two thermistors on either side of the sensor electrode were connected to the Wheatstone bridge circuit, respectively, and 1.2 V of voltage was applied to the microheater. This experiment used nitrogen gas and a gas generator to control the flow rate, and the gas pipe was connected to the tubing containing the micro flow sensor. The flow rates of the N_2_ gas (in sccm (cm/s)) in the tube were controlled to 25.65 (5), 51.3 (10), 77 (15), 102.6 (20), 128 (25), 154 (30), 179.5 (35), and 205.2 sccm (40 cm/s) (the numerical value in parentheses indicates the velocity of the fluid inside the tubing). A signal monitoring system performed real-time checks on the output voltage, whereas nitrogen (N_2_) gas flowed at a specific flow rate from the gas generator to the micro flow sensor.

**Figure 7:**
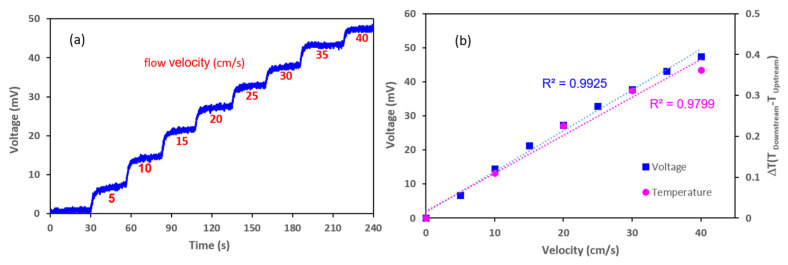
Results of the gas flow test using the flexible micro thermal flow sensor. (**a**) Graph showing voltage value for gas flow rate over time. (**b**) Graph comparing the temperature difference (the simulation result) with the voltage value against the flow rate.

As the flow rate varied between 5 and 40 cm/s, the change in resistance resulting from the temperature difference between the two thermistors (located on either side of the microheater) was converted into a voltage change. The results are presented in Figure 7. Figure 7a contains a stair-like graph of increasing flow rate over time. A graph showing the voltage value against the flow rate was derived using the average voltage value from each flow rate range, and a simulation result for the gas flow was plotted in Figure 7b for reference. The result of the gas flow measurements for the coefficient of determination of the graph for the change in the voltage against a flow rate between 5 and 40 cm/s was 0.99, which demonstrates an excellent linearity. For each flow rate range, the average standard deviation of the values was 0.38 mV; as the flow rate increased by 1 cm/s, the voltage increased by 1.2 mV. The maximum noise signal was 0.77 mV, and a resolution of 0.64 cm/s was achieved in the flow rate.

### 3.3. MEMS Flow Sensor Test in Continuous Liquid Flow

**Figure 8:**
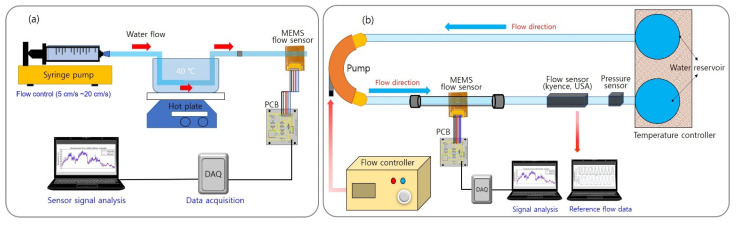
Schematics of the experimental setups for (**a**) the continuous liquid flow experiment using a syringe pump and (**b**) the pulsating liquid flow experiment using an artificial heartbeat model.

**Figure 9:**
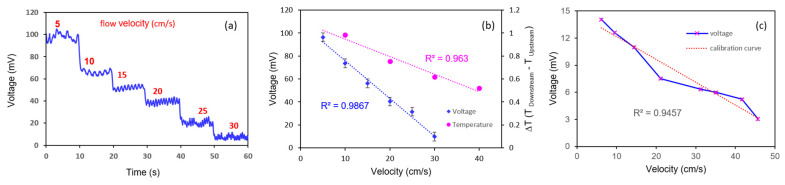
Results of the liquid flow test using the flexible microthermal flow sensor. (**a**) Graph showing the voltage value for liquid flow rate over time. (**b**) Graph showing the voltage value and the temperature difference (i.e., the simulation result) against the liquid flow velocity. (**c**) Graph showing output voltage value of the MEMS flow sensor in accordance with the flow velocity in the artificial heartbeat model.

The sensor’s performance was analyzed in a continuous liquid flow system using a syringe pump (KDS 200; KD Scientific, Holliston, MA, USA). A 40 mm diameter syringe with a capacity of 200 mL was mounted on the syringe pump, and a 3 mm diameter tube was connected to the syringe. The micro flow sensor was inserted in the middle of the tube in the perpendicular direction of the flow—the sensor electrodes meet the fluid in the order of an upstream sensor, the heater, and a downstream sensor—and was sealed tightly to prevent leakage. A water temperature of 30–40 °C was maintained throughout the experiment. Figure 8a,b illustrates the experimental setups for analyzing the flow of a liquid using a syringe pump and an artificial heartbeat model, respectively. The correlation between the output voltage generated from the sensor within the specific flow rate range and the fluid flow rate generated by the system was determined by adjusting the flow rate to achieve the desired speed within the tube containing the sensor. Within the tube, the flow rate was controlled to between 5 and 30 cm/s, and the sensor’s output voltage was measured within the controlled flow. The two thermistors and the microheater were connected to the PCB, which included the Wheatstone bridge circuit, and the output value was adjusted to zero by controlling the variable resistance inserted in the PCB under the initial flow. Then, the output voltage generated according to the fluid flow rate was monitored. It was revealed that as the flow rate increased, the output voltage decreased accordingly. Figure 9a shows the change in the output voltage of the sensor with varying flow rates over time. Figure 9b presents a graph showing the output voltage value of the sensor according to the flow rate of the fluid with the liquid flow simulation rate for comparison. At over 0.98, the coefficient of determination for the voltage value against the flow rate revealed a good linearity. Despite maintaining the same flow rate for over 10 s, the voltage value remained constant. The results of this experiment reveal that an increase in flow rate of 1 cm/s caused an average decrease of 3.6 mV in the output voltage; furthermore, the average standard deviation of each flow rate section was ±2 mV, which indicates a sufficient resolution of 2 cm/s or less.

### 3.4. Results of the Liquid Flow Experiment Using an Artificial Heartbeat Model

To evaluate the performance of the flow sensor in a liquid flow under constant pulsation (rather than under only a constant flow), this experiment used a cardiovascular model to simulate the heartbeat movement of a living body. The cardiovascular model comprised two water reservoirs, a pump, 3 mm diameter tubing (to replicate blood vessels), and a signal control system. A commercial flow sensor (FD-XS1; Keyence, USA) was inserted in the center of the tube to measure the real-time flow rate, and the MEMS flow sensor was equipped with the same line as the commercial sensor. The control system of the cardiovascular model allowed the beat rate, relative pressure, and stroke to be adjusted to provide a linear increase in flow rate with increases in relative pressure and stroke. The parameters for this experiment included a beat rate of 40 bpm and an internal flow rate velocity range of 5–40 cm/s; the data from the MEMS flow sensor were obtained within this range. Figure 9c shows the output voltage value of the MEMS flow sensor according to the flow rate produced by the artificial heartbeat. This experiment found that when the fluid velocity increased by 1 cm/s, the voltage decreased by an average of 0.36 mV. The average standard deviation in each flow rate range was 0.09 mV. The flexible MEMS flow sensor fabricated in this study was confirmed to realize a resolution of 0.5 cm/s for a pulsating liquid flow. The resolution was different between continuous flows and pulsatile flows in this study, and it is thought that the high level of noise signal in the continuous flow reduced the resolution. Table 2 summarizes the performance of the flexible MEMS flow sensors in the gas and liquid flow tests.

## 4. Conclusions

This study designed and fabricated a flexible microthermal flow sensor using MEMS technology. A CFD analysis provided the basis for the design of the sensor electrode, and a sensor module capable of measuring fluid flow signals was fabricated along with an electrical circuit system. LabVIEW software was used for the real-time monitoring of the output voltage signals from the sensor. The fabricated flow sensor module was confirmed to measure both liquid and gas flows; furthermore, its successful operation under a continuous liquid flow (with a syringe pump) and a pulsating flow (with an artificial heartbeat model) was verified. This study’s fabricated sensor can operate with a variety of fluids and can measure a wide range of flow rates. It is anticipated that the flexible micro flow sensor technology developed in this study can be applied in various industrial fields. Although some adaptations to its electrical packaging are required, the sensor has significant potential for application in healthcare fields, particularly for the measurement of biosignals, including respiration and the flows of body fluids.

## Figures and Tables

**Table 1:** The boundary conditions and the material properties used in the flow simulation.

Dimension (mm)		Materials				Boundary Condition		
			Air	Blood	Platinum			
Flow path	1 × 1 × 3	Density (kg/m^3^)	1.225	1060	21450	Heater	Temperature	314 k
Heater	0.3 × 1	Specific Heat (j/kg·k)	1006	3762	134	RTD1,2	Thermal condition	Heat Flux
							Shell conduction	1 Layer (Thickness (m): 2 × 10^−5^)
RTD 1	0.2 × 1	Thermal Conductivity (w/m·k)	0.0242	0.52	69.1	Inlet	Velocity (m/s)	0.1~0.4
							Temperature	310 k
RTD 2	0.2 × 1	Viscosity (kg/m·s)	1.79 × 10^−5^	0.00278		Outlet	Temperature	310 k

**Table 2:** Performance indicators for gas and liquid flows using flexible MEMS flow sensors.

	Gas	Liquid	
		Continuous Flow	Pulsating Flow
**Temperature** **(** **Δ** **T, simulation)**	0.1 °C ↑/10 cm/s	0.2 °C ↓/10 cm/s	
**Voltage**	1.2 mV/cm ± 0.4 mV	3.6 mV/cm ± 3.7 mV	0.36 mV/cm ± 0.12 mV
**Resolution**	0.64 cm/s	1.1 cm/s	0.5 cm/s
**Noise level**	0.77 mV	4 mV	0.18 mV
**Range**	0~40 cm/s	0~30 cm/s	0~40 cm/s
**Power consumption**	40 mW		

## Data Availability

Please contact the corresponding author for data requests.
